# Recruiting Asian Americans for Online Studies: Methodological Systematic Review

**DOI:** 10.2196/71765

**Published:** 2025-07-03

**Authors:** Fang Lei, Fei-fei Huang, Ying Jiang

**Affiliations:** 1 School of Nursing, University of Minnesota Twin Cities Minneapolis, MN United States; 2 School of Nursing, Fujian Medical University Fuzhou China; 3 Alice Lee Centre for Nursing Studies, Yong Loo Lin School of Medicine, National University of Singapore Singapore Singapore

**Keywords:** Asian American, methodology, online study, recruitment, systematic review

## Abstract

**Background:**

Engaging Asian American participants in research studies helps to understand their health needs and health disparities better. However, recruiting Asian Americans for online studies remains challenging.

**Objective:**

This study aims to synthesize strategies for recruiting Asian Americans to research studies that collected data online and to further explore the characteristics of the recruited participants.

**Methods:**

A systematic review method was used. Data were searched in the PubMed, CINAHL, and OVID databases using Medical Subject Headings (MeSH) terms and title search strategies. A narrative synthesis was conducted to summarize strategies for recruiting Asian Americans to online research studies. An independent Student t test (2-tailed and unpaired) was performed to compare the characteristics of recruited Asian Americans, using SPSS 29.0 software. The study was reported in accordance with the PRISMA (Preferred Reporting Items for Systematic Reviews and Meta-Analyses) 2020 checklist.

**Results:**

After data extraction, 18 studies were included in this review. Results showed that strategies for recruiting Asian Americans to research studies that collected data online included both online and offline methods. Online recruitment methods included online survey market services, social media groups, online advertisements, and email lists. Offline recruitment methods included churches, community organizations, local clinics, health care centers, American Cancer Society local chapters, and cancer registries. Among the online and offline recruitment methods, social media groups and community recruitment were the most frequently used, respectively. The most commonly used online study platform was self-designed project websites. Participants engaged in the online studies tended to be in their middle adulthood and have a high level of education beyond high school. Compared with those recruited offline, participants recruited online tended to be younger and more highly educated.

**Conclusions:**

This study suggests that researchers may use mixed recruitment methods, combining both online and offline approaches, to recruit Asian Americans to online studies. When selecting the recruitment venue, researchers should consider project budget, data security, data quality, and credibility. They should also be aware of the distinct characteristics of participants recruited online versus offline.

## Introduction

Asian Americans are defined as “Americans who have origins in any of the original peoples of East Asia, Southeast Asia, or the Indian subcontinent” [1]. They are the fastest-growing racial group in the United States [2]. In 2020, it was reported that there were more than 24 million Asian Americans in the United States, comprising 7.2% of the total population [3]. With this number continuing to grow, Asian Americans are estimated to reach 41 million by 2050, accounting for 9% of the US population [4].

Asian Americans include several subgroups. In 2019, the 6 major origin groups among Asian Americans were Chinese (5.4 million, 24%), Indian (4.6 million, 21%), Filipino (4.2 million, 19%), Vietnamese (2.2 million, 10%), Korean (1.9 million, 9%), and Japanese (1.5 million, 7%), together accounting for 90% of all Asian Americans [2]. The remaining Asian American (10%) subgroups each made up about 2% or less of the total Asian American population [2].

Although the number of Asian Americans is growing, they remain one of the most understudied racial minority groups [5]. According to research studies recorded in MEDLINE/PubMed, only 0.01% of articles published between 1966 and 2000 included Asian American participants [5]. Additionally, only 0.17% of the National Institutes of Health–funded clinical research budget between 1992 and 2018 was allocated to studies involving Asian Americans [5]. Given the health disparities observed in Asian Americans, such as higher rates of specific cancers, heart disease, and other chronic conditions compared with their White counterparts [6], it is essential to engage Asian American participants in research studies to better understand their health needs and develop effective, tailored interventions.

In recent years, researchers have conducted multiple studies on Asian American populations, although this group is less willing than other racial groups to participate in health research [7]. Researchers have encountered difficulties in recruiting and retaining Asian American participants in their studies [8]. Multiple barriers to recruiting minority populations, including Asian Americans, have been identified in previous studies [9]. These barriers exist at 3 levels: institutional, researcher, and participant levels [10]. Institutional-level barriers include provider time constraints and competing service demands. Researcher-level barriers include multicultural differences, lack of knowledge, and bias against research. Participant-level barriers include distrust of research, concerns about confidentiality, fear for safety, scheduling conflicts, limited access to medical care, lack of knowledge, language difficulties, and cultural differences [10].

Because of the scarcity of Asian Americans in many states in the United States (eg, North Dakota, South Dakota, Montana) [3], it is difficult to collect sufficient data from Asian American participants in research studies. This also makes it challenging for researchers to conduct local, in-person studies involving Asian Americans. As a result, online data collection has become an alternative method for reaching more Asian Americans at the national level for survey-based studies. Despite this, researchers who have conducted online studies with Asian Americans have reported certain challenges in recruitment. For example, internet-recruited samples tended to be younger, US-born, fluent in English, and more educated compared with participants recruited offline [11,12]. Furthermore, researchers have identified several additional issues in their online recruitment of Asian Americans, including difficulties in establishing trustworthy relationships with participants, concerns about breaches of personal information over the internet [13], and challenges in maintaining rapport with gatekeeper recruiters, such as Facebook group moderators and Asian American community directors [14].

Recruitment of minority research participants poses unique challenges compared with recruitment among the general population [11]. Before the rise of internet-based research, studies commonly used methods such as in-person and telephone surveys to enhance Asian American participation. One notable approach was surname-based sampling, which has been used to identify individuals of Asian descent in both clinical and population-based studies [15,16]. For example, Kim et al [17] revisited the efficacy of the “Kim” sampling method across Korea and the United States, highlighting both the potential and limitations of this strategy. These early methodological efforts provide valuable context for evaluating contemporary online recruitment practices and underscore the continued need for culturally and linguistically tailored sampling strategies.

Although, to our knowledge, there are no systematic reviews specifically focused on recruitment strategies for Asian Americans in online studies, a limited number of studies have incidentally reported on recruitment approaches when including Asian American populations in health-related research. While recruitment may not have been the primary focus of these studies, they often provide relevant insights that, when synthesized, can offer valuable guidance for researchers seeking to engage Asian American participants in online research. Given the rapid expansion of digital health research, the scarcity of available evidence, and the urgent need to address this gap, this study aims to synthesize strategies for recruiting Asian Americans in studies that collect data online and to further examine the characteristics of recruited participants. This study will provide important information to researchers regarding recruitment methods, online study platforms, and the characteristics of Asian Americans participating in online studies—ultimately helping to optimize recruitment efforts for this population.

## Methods

### Research Questions

The research questions proposed to address the study aim were as follows: (1) What methods were used to recruit Asian Americans to online studies? (2) What platforms were used in studies that collected data online from Asian Americans? (3) What were the characteristics of the Asian American participants recruited for these online studies?

The target population (P) for the research question was Asian Americans. The primary outcome (O) explored in this study was the recruitment methods used. Additionally, the study examined other outcomes, including the online data collection platforms utilized and the characteristics of the Asian Americans recruited to studies that collected data online.

### Design

This study used a conventional systematic review design and was reported in accordance with the PRISMA (Preferred Reporting Items for Systematic Reviews and Meta-Analyses) 2020 checklist (Multimedia Appendix 1). It is not a living systematic review, and no regular update schedule or version control was implemented.

### Inclusion and Exclusion Criteria

The inclusion criteria for studies in this systematic review were as follows: (1) primary studies that provided information on the recruitment of Asian Americans for studies in which data were collected online. This included studies that recruited participants either offline, online, or both, as long as the data collection process was conducted online; (2) studies in which the research population consisted solely of Asian Americans; and (3) studies published in English. We included studies conducted in the United States that focused on the recruitment of Asian American participants, regardless of study design.

The exclusion criteria were as follows: (1) studies in which data were collected offline; (2) studies with recruitment efforts targeting the general population rather than specifically focusing on Asian Americans, including approaches that may not be adaptable to the Asian American population [11]; (3) secondary studies that did not provide information on participant recruitment or online study implementation; and (4) studies lacking data support, such as commentaries, study protocols, or other nonempirical reports.

### Data Sources and Search Strategies

The research team consisted of 3 nurse researchers with expertise in cross-cultural research, survey methodology, and systematic reviews. We had substantial research experience working with Asian American populations and were well-versed in the systematic review process. Additionally, we consulted with a university-affiliated health sciences librarian who had expertise in systematic review methodology. The librarian assisted in refining search terms, selecting appropriate databases, and ensuring the comprehensiveness and reproducibility of the search strategy.

We searched for studies published before February 22, 2024, in the PubMed, CINAHL, and OVID databases using Medical Subject Headings (MeSH) terms and title-based search strategies. Our goal was to identify all relevant studies that could help answer our research questions. The search strategies used for each database are listed in Textbox 1.

In addition to database searching, we conducted manual searches of the reference lists of eligible studies to identify further relevant articles. See Multimedia Appendix 2 for the full search strategy.

**Search strategies.**

**1. PubMed**

The MESH term search strategy was applied. We used the MESH terms (internet) AND (Asian Americans) to advanced search relevant studies.

**2. CINAHL**

The title search strategy was applied. We used (TI internet OR TI web-based OR online) AND (TI Asian Americans OR TI Asian American) to search.

**3. OVID**

The title search strategy was applied. We used (TI internet OR TI web-based OR online) AND (TI Asian Americans OR TI Asian American) to search.

### Selection Process and Data Extraction

We used 3 databases—PubMed, CINAHL, and OVID—to triangulate and supplement our search results. Articles retrieved from these databases were screened by title, abstract, and full text. The inclusion and exclusion criteria were applied during the screening and data extraction processes. Zotero (Corporation for Digital Scholarship) reference management software was used to organize studies that met the criteria. Zotero’s built-in duplicate detection feature, which identifies records based on title, author(s), and publication year, was used to detect duplicates. All identified duplicates were manually reviewed to ensure accuracy. When duplicates were confirmed, 1 version from each duplicate set was retained, and the others were removed. Full texts of the articles were then reviewed to extract relevant information. A table of evidence was created to capture key details from each study, including citation, purpose, design, sample, recruitment method, and online study platform. Two researchers (FL and FFH) independently conducted the literature search and extracted data into the table of evidence based on the predetermined criteria. Any discrepancies between the 2 reviewers regarding extracted data or study inclusion were identified by comparing the independently completed tables and were resolved through discussion and consensus. When consensus could not be reached, a third reviewer (YJ) was consulted to adjudicate. This process ensured methodological rigor and helped minimize bias during the data extraction stage.

### Data Analysis

The quality of the included studies was independently assessed by the same 2 reviewers (FL and FFH) who conducted the article screening. Any disagreements were resolved through discussion, and a third reviewer (YJ) was consulted if consensus could not be reached. Study quality was evaluated using appropriate tools based on study design, including the LEGEND Evidence Evaluation Tools for cross-sectional survey studies (Multimedia Appendix 3), the JBI (Joanna Briggs Institute) Checklist for Quasi-Experimental Studies (Multimedia Appendix 4), the CASP (Critical Appraisal Skills Programme) Randomized Controlled Trial Appraisal Tool (Multimedia Appendix 5), and the CASP Qualitative Studies Assessment Tool (Multimedia Appendix 6). These tools were selected because they are widely used in systematic reviews to assess observational, interventional, and qualitative research, offering a standardized and structured approach to evaluating key aspects such as study design, sample selection, data collection, and reporting. The domains covered by these tools aligned well with the types of studies included in our review and enabled us to assess both the methodological rigor and the relevance of each study to our research question.

Because of the heterogeneity of study designs included in this review (eg, observational, quasi-experimental, randomized controlled, and qualitative studies), a formal GRADE (Grading of Recommendations Assessment, Development and Evaluation) assessment was not feasible. However, core GRADE principles were applied to qualitatively assess the overall certainty of the cumulative evidence. This assessment included consideration of study limitations (risk of bias), consistency of results across studies, directness of the evidence in relation to the review question, and overall coherence of the findings. These criteria were used to guide a narrative judgment of the overall certainty of the evidence (eg, high, moderate, or low).

The extracted data were synthesized using a comparative analysis approach. Differences in the characteristics of participants recruited online versus offline were further analyzed using an independent Student *t* test (2-tailed and unpaired), conducted with SPSS version 29.0 (IBM Corp). The decision not to perform a formal meta-analysis was based on substantial heterogeneity among the included studies in terms of study design (eg, cross-sectional, quasi-experimental, randomized controlled trial, and qualitative), participant characteristics, outcome measures, and online intervention modalities. The variability in outcome definitions, measurement tools, and data reporting formats made it inappropriate to statistically pool results across studies without introducing significant bias or risk of misinterpretation. Given these methodological differences, a meta-analysis would have lacked validity. Therefore, a narrative synthesis, supported by descriptive and comparative statistics, was deemed more appropriate for this review.

### Ethical Considerations

This is a secondary systematic review study. Ethical approval is not needed for the research of this study design.

## Results

### Study Characteristics

**Figure 1:**
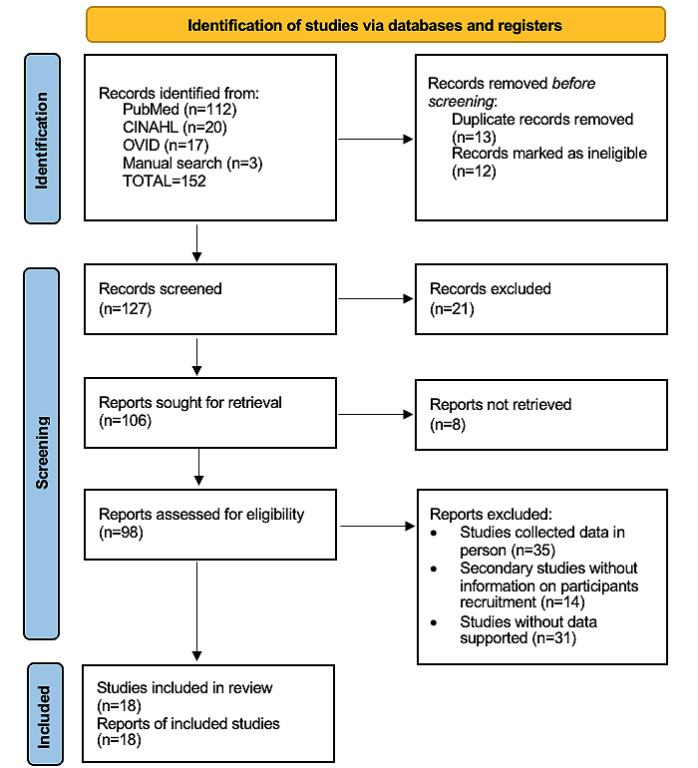
PRISMA (Preferred Reporting Items for Systematic Reviews and Meta-Analyses) flowchart.

A total of 152 studies were initially identified through the literature search. After removing duplicates and excluding ineligible studies, 18 studies were included in the final review, as illustrated in Figure 1 and summarized in Table 1. Of these, 9 were cross-sectional survey studies [18-26], 1 was a pre- and post–quasi-experimental intervention study [27], 6 were randomized controlled trials [28-33], and 2 were qualitative studies [34,35]. Sample sizes ranged from 17 [34] to 3084 [24], and the publication years spanned from 2005 [19] to 2024 [27].

The reviewed studies addressed a broad range of health-related and sociocultural issues affecting Asian Americans, including topics such as cancer survivorship, physical activity, discrimination, organ donation, and masculinity norms. The studies included in this review can be broadly categorized into 5 thematic clusters: (1) cancer-related studies (n=3), which focused on survivorship [28,35] and supportive care needs [27]; (2) menopause and midlife health (n=6), explored primarily through a series of studies led by Im et al [19,20,32-34] and Chee et al [29], which examined menopausal symptom experiences, attitudes toward physical activity, and online coaching programs tailored for Asian American midlife women; (3) discrimination, racism, and mental health (n=4), investigated during the COVID-19 pandemic [21,23,25,26], focusing on the psychological impacts of racism and the moderating effects of ethnic identity and online behavior; (4) substance use prevention in youth (n=2), addressed by Fang et al [30,31] through family-based online interventions aimed at reducing substance use among Asian American adolescent girls and strengthening parent-child protective factors; and (5) health behavior and public health engagement (n=3), which examined attitudes toward organ donation [18], skin cancer screening [22], and grocery shopping behaviors [24], highlighting cultural and systemic barriers. All studies were led by US-based researchers and conducted within the United States, reflecting a strong domestic interest in addressing health disparities among Asian American populations. Notably, 8 of the 18 (44%) studies [18,21,23-27,29] were published within the past 5 years (2020-2024), indicating a growing academic interest in using online methodologies to study health issues in Asian Americans—particularly in response to the COVID-19 pandemic, when online data collection became essential.

**Table 1:** Characteristics of the included studies.

Citation	Purpose	Design	Sample	Recruitment Method	Online study platform
Alolod et al [18]	To explore Asian American’s willingness to donate their own and family members’ organs and tissue upon death. The predominant role of the family is also explored, and key associations between donation disposition and sociodemographic characteristics are identified.	An online survey study design	Asian American population (n=899)	Between June and October 2019, an online market research panel hosted and recruited through Qualtrics, Inc was utilized to target a random and nationally representative sample of adults, who self-identified as Asian American. Participants were compensated directly by Qualtrics based on a format they chose during panel enrollment with the company and earned points for various rewards systems, such as shopping or travel points.	Qualtrics
Im et al [19]	To describe menopausal symptoms, perceived causes of the symptoms, and management strategies for the symptoms among 5 ethnic groups of Asian American women	A cross-sectional descriptive internet survey design	University faculty or staff members aged 40-60 years who self-reported as Asian Americans and could read and write English, missing responses less than 10%, first-generation immigrants to the United States, self-reported Asian Americans, and ability to read and write in English (n=62)	The study was announced through the project home page and email lists provided by the websites of the universities. The websites/pages of the first 300 universities from among the 1476 US universities generated by Yahoo! and AltaVista were visited. Faculty and staff members’ email addresses were obtained through the universities’ websites/pages.	Self-designed project website
Im et al [20]	To compare the menopausal symptom experiences of subethnic groups of Asian American midlife women	A cross-sectional study design	Asian American women (n=91)	ICAAs^a^ were also targeted for the posting of the study announcement because studies reported that ethnic minorities were more successfully recruited in churches and support/social groups that have ethnic and culturally specific memberships. Participants were recruited by making announcements about the study in ICMWs^b^ and ICEMs^c^.	Self-designed project website
Le et al [21]	To investigate 3 main research questions: (1) the associations between racism and gendered racism and muscularity-oriented disordered eating; (2) the associations between conformity to distinct masculine norms and muscularity-oriented disordered eating; and (3) if ethnic identity moderates the association between racism and gendered racism and muscularity-oriented disordered eating.	An online survey study design	Asian American men aged 18 years and over, identification as an Asian American man, and current residence in the United States (n=220)	The participants were recruited through a variety of online distribution channels. These channels included posting recruitment messages to various email lists, social media sites, and public online forums that serve the Asian American community. Examples include Asian cultural organizations at various universities across the United States as well as the popular social site Reddit.	No details about this information
Lingala et al [22]	To examine factors that correlate with “ever having skin checked by a dermatologist” in a Northern California Asian American population	An online survey study design	Adults in Northern California who self-identified as Asian American (n=469)	The website address was distributed to leaders of Northern California community groups that were likely to contain a large portion of Asian Americans for circulation among community group members to identify participants for this study.	The survey was placed online via the Stanford University Surveyor website.
Pan et al [23]	Three discrimination-related variables were studied, including experience of discrimination, worry about discrimination, and racism-related social media use during the COVID-19 pandemic among Asians in the United States. Relationships of these 3 variables related to depression were examined. How the association between racism-related social media use and depression was moderated by personal experience of and worry about racial discrimination was also examined.	A web-based, cross-sectional survey	People who identified themselves as Asian and resided in the United States (n=209)	Amazon Mechanical Turk	The survey was first created on Qualtrics and then distributed to qualified participants via Amazon Mechanical Turk.
Rummo et al [24]	To examine differences in online grocery shopping behaviors among Asian American adults using data disaggregated by Asian American ethnic subgroup and levels of acculturation	An online survey study design	A nationally derived nonprobability sample of Asian American adults. Eligibility criteria were identifying as Asian, being 18 years of age or older, and being able to read and speak English (n=3084)	The participants were recruited using Dynata, an online surveying company that recruits volunteer research participants.	Open REDCap^d^, an online survey platform, was used to create and distribute the survey.
Yang et al [25]	To explore whether experience with discrimination was associated with more social media use among Asian people and how adaptive social media use was for their well-being during COVID-19	A descriptive online survey study design	Asians/Asian Americans residing in the United States (n=242)	Recruited from Amazon Mechanical Turk	Amazon Mechanical Turk
Yu et al [26]	To examine the types of discrimination and worries experienced by Asians and Asian Americans living in the United States during the pandemic, as well as factors that were associated with everyday discrimination experience and concerns about future discrimination that the Asian community may face	A cross-sectional online survey design	Eligible participants included individuals aged ≥18 years who identified as Asian and lived in the United States when the data were collected (n=235)	Amazon Mechanical Turk	The survey was first created on Qualtrics and then distributed to qualified participants via Amazon Mechanical Turk
Wang et al [27]	To examine supportive care needs in 4 major domains (cancer information, daily living, behavioral health, and language) and participant factors correlated with these needs; identifying profiles of supportive care needs; and examining whether the identified needs profiles are associated with quality of life.	An online intervention study	The inclusion criteria were as follows: self‐identified as Asian or Asian American; aged 21 years or older; spoke English, Cantonese, Mandarin, or Vietnamese; had stage I-IV colon, rectum, liver, or lung cancer; received health care in 1 of 9 Greater Bay Area counties; were currently receiving or planning to receive treatment; had access to or were willing to create an email account; and were willing to stay in the study for 6 months. The exclusion criteria were any medical or psychological conditions precluding informed consent, receiving institutionalized care (eg, assisted living, hospice, and incarceration), or if the patient already completed treatment (n=47)	Participants were identified using an early case ascertainment process from the Greater Bay Area Cancer Registry. The Greater Bay Area Cancer Registry is a population‐based cancer registry that covers 9 counties in the San Francisco Bay Area of California and is part of the National Cancer Institute’s Surveillance, Epidemiology, and End Results program and the statewide California Cancer Registry.	The portal was built on Salesforce, a secure, HIPAA^e^‐compliant cloud‐based platform.
Chee et al [28]	To pilot-test the culturally tailored ICSG-AA^f^ for breast cancer survivors and determine the preliminary efficacy of the ICSG-AA in enhancing the women’s breast cancer survivorship experience	A randomized repeated measures pretest/posttest control group design	Self-reported women aged 21 years and older who had a breast cancer diagnosis in the past 5 years; could read and write English, Mandarin Chinese, Korean, or Japanese; had access to the internet; and identified their subethnicity as Chinese, Korean, or Japanese (n=65)	The participants were recruited through the internet and physical settings where Asian American breast cancer survivors gathered (eg, internet communities for Asian American breast cancer survivors, Facebook sites, local clinics, American Cancer Society local chapters).	Self-designed project website
Chee et al [29]	To determine the preliminary efficacy of a newly developed online program for physical activity promotion on cardiovascular symptoms of Asian American midlife women.	A pilot repeated measures randomized controlled trial (pretest/posttest)	Asian American (Chinese or Korean) women who were 40-60 years old; self-identified as Chinese or Korean residing in the United States; could speak English, Mandarin Chinese, or Korean; and were able to utilize mobile devices or computers (n=91)	Participants were recruited via online communities and groups for Chinese or Korean Americans in the United States, identified through search engines. The first 10 eligible communities or groups were contacted for study announcements, and each week, an additional 10 communities or groups were contacted.	No details about this information
Fang et al [30]	To examine the efficacy and generalizability of a family-oriented, web-based substance use prevention program for young Asian American adolescent girls	Randomized controlled trial, intervention-arm dyads completed a 9-session web-based substance use prevention program	Asian American girls aged 10-14 years and their mothers. Girls needed to be Asian, be aged between 11 and 14 years, have private access to a computer, and have mothers’ active participation (n=108)	The participants were recruited through online advertisements and from community service agencies. Study participants were recruited through advertisements on Craigslist and in mailings to Asian community service agencies in 19 states with significant Asian populations.	No details about this information
Fang et al [31]	To evaluate the parent-child program’s efficacy in decreasing girls’ substance use, and modifying risk and protective factors at individual, family, and peer levels	Randomized controlled trial; intervention-arm dyads completed a 9-session web-based substance use prevention program	Asian American adolescent girls aged 10-14 years and their mothers, randomly assigned to an intervention arm or to a test-only control arm (n=108)	Asian American mother-daughter dyads were recruited through online advertisements and from community service agencies. The participants were recruited from 19 states in the United States via postings on social network sites and advertising through social service agencies.	No details about this information
Im et al [32]	To test the efficacy of a technology-based information and coaching/support program on menopausal symptoms of Asian American breast cancer survivors	A randomized repeated measures pretest/posttest control group design	Asian American breast cancer survivors, who identified themselves as Chinese, Korean, or Japanese; were aged 21 years and older; were diagnosed with breast cancer in the past 5 years; could read and write English, Mandarin Chinese, Korean, or Japanese; and could access the internet (n=91)	The participants have been recruited through online and offline support/social groups for Asian Americans (eg, churches, organizations, forums, health care centers, and professional groups). Study announcements were made by sending messages to gatekeepers (eg, website owners and pastors) and by posting study flyers in various groups (eg, social media groups and community support groups).	Self-designed project website
Im et al [33]	To determine the efficacy of the web-based program in improving menopausal symptom experience of Asian American midlife women	A pilot randomized controlled trial online intervention study design	Self-reported Chinese or Korean American midlife women aged 40-60 years, whose parents and grandparents were Chinese or Korean; who were able to read and write English, Mandarin Chinese, or Korean; who were living in the United States; and who had access to computers, smartphones, or tablets (n=29)	The recruitment settings included online communities for Chinese and Korean Americans. Online searches were performed through Google, Yahoo!, and Bing, and the online communities for Chinese or Korean Americans were identified/verified. Then, all the online communities on the verified list were contacted for study announcements.	Self-designed project website
Im et al [34]	To explore Asian American midlife women’s attitudes toward physical activity using a feminist perspective	A qualitative online forum study design	Midlife Asian American women who were aged 40-60 years; were ambulatory and able to participate in all forms of physical activity; could read and write English; and had access to the internet. Those who had high cardiovascular and musculoskeletal risk factors such as major signs or symptoms suggestive of pulmonary or cardiovascular disease; a history of a myocardial infarction, stroke, or type I diabetes mellitus; blood pressure higher than 160/100 mm Hg; and use of beta-blockers, diltiazem, or verapamil were excluded from the study (n=17)	The participants were recruited through the internet using a convenience sampling method. The study announcement was made through both ICMWs and ICEMs.	Self-designed project website
Im et al [35]	To explore how Asian Americans living with cancer who participated in ICSGs viewed ICSGs, what facilitated or inhibited their participation in ICSGs, and what cultural values and beliefs influenced their participation in ICSGs	A 1-month qualitative online forum design	Asian American patients with cancer recruited through a convenience sampling method, Asian Americans living with cancer that could read and write English (n=18)	To recruit the participants, 2 types of recruitment sites were approached: (1) ICSGs and (2) ICAAs. These sites were searched for on Google, and a total of 210 ICSGs and ICAAs were retrieved. All the administrators of the retrieved ICSGs and ICAAs were contacted and asked to post an announcement regarding the study. A total of 15 administrators agreed to post an announcement on their website; the others did not respond to the contact emails.	Self-designed project website

^a^ICAA: Internet Communities for Asian Americans.

^b^ICMW: Internet Communities for Midlife Women.

^c^ICEM: Internet Communities for Ethnic Minorities.

^d^REDCap: Research Electronic Data Capture.

^e^HIPAA: Health Insurance Portability and Accountability Act.

^f^ICSG-AA: Internet Cancer Support Groups-Asian Americans.

### Study Quality

The LEGEND Evidence Evaluation Tools for cross-sectional survey studies indicated that the included cross-sectional studies demonstrated good quality in terms of validity and reliability. These tools were selected for their suitability and alignment with the observational study designs commonly used in health research. The applicability of the studies could not be evaluated, as the assessment questions were not relevant to the context of this review (eg, “Are my patient’s and family’s values and preferences satisfied by the knowledge gained from this study?” and “Would you include this study/article in the development of a care recommendation?”).

The JBI Checklist for Quasi-Experimental Studies indicated that the pre- and post–quasi-experimental intervention study [27] was of medium quality, with a score of 5 out of 9 (56%). According to criteria used in previous systematic reviews, studies with a JBI score above 70% are classified as high quality, those scoring between 50% and 70% as medium quality, and those below 50% as low quality [36].

The CASP-Randomized Controlled Trial Appraisal Tool indicated that the design, methodology, and results of the included randomized controlled trials were of acceptable quality, based on quality score cut-offs suggested by previous researchers [37]. However, the applicability of the study results could not be assessed, as certain appraisal questions were not relevant to the context of this review (eg, “Would the experimental intervention provide greater value to the people in your care than any of the existing interventions?”).

The CASP-Qualitative Studies assessment tool indicated that both included qualitative studies [34,35] were of good quality, each receiving a score of 9 out of 10. In general, higher CASP scores reflect stronger methodological rigor, with scores above 6 suggesting good quality. By contrast, lower scores indicate potential limitations in research design or execution, with scores of 3 or below considered poor, and scores between 4 and 5 categorized as fair [38].

To synthesize the risk of bias across studies, we evaluated recurring methodological limitations identified in the included studies. The most common sources of potential bias were reporting bias in cross-sectional surveys (stemming from self-reported outcomes), selection bias in the quasi-experimental study (due to the absence of randomization), and performance bias in some intervention studies (resulting from the lack of blinding). However, the qualitative studies demonstrated strong methodological rigor, with minimal indications of bias. Based on the GRADE principles, the overall body of evidence was assessed as having moderate certainty. This rating reflects some concerns regarding methodological limitations, while also recognizing the consistency of findings across studies and their direct relevance to the review question.

### Recruitment Methods

#### Overview

The methods for recruiting Asian Americans to studies that collected data online can be categorized into online and offline recruitment approaches. Among the 18 included studies, 12 used online recruitment methods exclusively [18-21,23-26,29,33-35], 4 used a combination of both online and offline recruitment methods [28,30-32], and 2 relied solely on offline recruitment methods [22,27].

#### Online Recruitment Methods

These included (1) online survey market services (n=5), which are tools or websites used to facilitate data collection; (2) social media groups (n=8), which are online spaces where users connect and share information; (3) online advertisements (n=3); and (4) email lists (n=2).

The commonly used online survey market services were the Qualtrics online research panel [18], the online surveying company Dynata [24], and Amazon Mechanical Turk (MTurk) [23,25,26].

Social media groups used to recruit Asian Americans for research studies included internet communities [20,28,29,32-35] such as the Internet Communities for Ethnic Minorities and the Internet Communities for Asian Americans [20,28,34,35]; online support or social groups [21,29,32]; and public online forums [21,32], such as Reddit [21]. Online social media groups were identified through search engines such as Google, Facebook, Yahoo!, and Bing. Once identified, recruitment posters, announcements, or website links were shared in the groups by either group administrators, group leaders, or the researchers themselves.

The online advertisement methods included promotions through Facebook pages [28] and the Craigslist website [30,31]. Participants received the consent or assent package by either postal mail or online, were assigned a unique study identification and password, and then completed the surveys or intervention modules online [28,30,31].

The email listing method involved using email lists obtained from the websites of target facilities [19,21], such as universities and cultural organizations relevant to the study population. These facilities were identified through search engines, including Yahoo! and Altavista. Once identified, recruitment messages were distributed to individuals on the email lists. Participants who agreed to participate were directed to the project website, where they received an information sheet about the study, provided informed consent, and completed the survey online.

#### Offline Recruitment Methods

Among the 18 studies, 6 used offline recruitment methods. These included recruiting participants through health care centers and churches [32], community organizations [22,30,31], local clinics and American Cancer Society local chapters [28], and cancer registries [27]. Recruitment messages or mailings were sent to the administrators of these facilities, or study flyers were posted on-site to invite participants.

### Online Study Platform

In this review, online study platforms were categorized into 2 types based on their origin and functionality: self-designed platforms and commercial platforms. A self-designed platform refers to a digital interface or system specifically developed by the research team or their affiliated institution to support the implementation of the study. These platforms were often custom-built to deliver a specific intervention, survey, or educational content, and were not accessible to individuals outside the study context. Technical features of self-designed platforms typically included unique programming, secure internal hosting, and limited scalability beyond the study population. By contrast, a commercial platform refers to an existing, widely available web-based or mobile platform developed and maintained by third-party vendors or companies.

Among the 18 studies, 8 utilized self-designed project websites to conduct their studies [19-21,28,32-35]. Four studies did not report information regarding the platforms used [21,29-31]. Three studies used both Qualtrics and Amazon MTurk for data collection [23,25,26]. The remaining 3 studies used Qualtrics [18]; REDCap (Research Electronic Data Capture) [24]; and Salesforce, a secure, HIPAA (Health Insurance Portability and Accountability Act)-compliant cloud-based platform, respectively [27].

### Characteristics of the Recruited Participants

Among the 18 included studies, the mean age of participants ranged from 23.19 years [21] to 57.6 years [27], with a median mean age of 41.72 years (Table 2). The proportion of participants with an education level above high school ranged from 68% [30] to 100% [19,21]. The percentage of participants who were employed ranged from 28.6% [21] to 95% [19]. Several studies [18-20,34,35] collected data on participants’ religious affiliations, with Catholicism, Protestantism, and Buddhism being the most commonly reported. Marital status was reported in 14 of the studies, while 4 studies [21,25,29,33] did not provide this information. Most of the participants recruited across the studies were married or partnered. The proportion of married or partnered individuals ranged from 40% [22] to 90.3% [20]. While all studies reported participants’ gender distribution, the use of diverse gender categories across studies made it infeasible to summarize gender distribution percentages in a consistent manner. We also attempted to collect data on participants’ household income, ethnicity distribution, immigration status, and residence status. However, due to either lack of reporting or inconsistent variable categorization across studies, further analysis of these characteristics was not feasible. Additionally, while comparing recruitment time and rates across different methods would have been valuable, insufficient reporting in the included studies precluded such analyses.

**Table 2:** Characteristics of the recruited participants.

Citation	Mean age (years)	Education level^a^, %	Occupation^b^, %	Ethnicity	Income
Alolod et al [18]	36	77.6	54.4	Chinese (n=204, 22.7%), Filipino (n=163, 18.1%), South Asian (n=139, 15.5%), Korean (n=72, 8.0%), Japanese (n=55, 6.1%), other Southeast Asian (n=173, 19.2%), and other Asian/multiethnic (n=93, 10.3%)	71.9% (n=646) reported annual household incomes above US $40,000
Im et al [19]	43.87	100	95	48% were Chinese, 18% Japanese, 11% Korean, 5% Taiwanese, and 10% Vietnamese	The mean annual income was US $60,445 (SD US $33,168.96); 52% reported their income as more than sufficient
Im et al [20]	49.4	85	56	34% were Chinese, 18% were Korean, 18% were Asian Indian, 12% were Filipino, and 19% were other Asians	Approximately half of the women (52%) said that it was not hard to pay for basic needs with their family income
Le et al [21]	23.19	100	28.6	32.7% Chinese, 15.2% Indian/South Asian, 11.5% Korean, 10.1% Vietnamese, 10.1% multiracial, and 20.4% other Asians	6% of the household income was under US $25,000; 14.3% household income was between US $25,000 and US $49,999; 79.7% household income was over US $50,000
Lingala et al [22]	37.2	92	N/A	N/A	68% more than US $50,000
Pan et al [23]	33.69	95.69	68.42	35.41% Chinese, others are non-Chinese	66.51% more than US $50,000
Rummo et al [24]	43.2	78.9	58.5	57.6% East Asian, 18.9% South Asian, 19.4% Southeast Asian	87.3% reported annual household income above US $20,000
Yang et al [25]	32.88	N/A	N/A	N/A	N/A
Yu et al [26]	32.87	94	67	34.8% Chinese, others non-Chinese	77.2% reported annual household income above US $40,000
Wang et al [27]	57.6	74.4	42.6	44.7% Chinese, others non-Chinese	72.4% reported annual household income above US $50,000
Chee et al [28]	47.0	75	54.8	45.2% Chinese, 19.4% Korean, 8.1% Japanese, and 25.8% other	The mean annual income was US $81,853 (SD US $59,904.4)
Chee et al [29]	46	92	65	69% Chinese and 31% Korean	81% of the women said that it was not hard to pay for basic needs with their family income
Fang et al [30]	40.24	68	N/A	N/A	N/A
Fang et al [31]	39.73	72.22	N/A	N/A	N/A
Im et al [32]	51.3	N/A	N/A	57% Chinese, 23% Korean, and 20% Japanese	48.31% of the participants reported that their yearly family income was sufficient or more than sufficient
Im et al [33]	45.7	93.1	69	72.4% Chinese and 27.6% Korean	82.8% of the participants reported that it was not hard to pay for basic needs with their family income
Im et al [34]	49.06	88.2	70.6	35.3% Chinese, 35.3% Korean, 17.6% Asian Indian, 5.9% Filipino, and 5.9% Sri Lankan	47.1% of the participants reported that it was not hard to pay for basic needs with their family income
Im et al [35]	39.89	100	66.7	44.4% Chinese, 5.6% Japanese, 11.1% Filipino, 11.1% Indian, 5.6% Persian, and 22.2% not specified	72.2% of the participants reported that their yearly family income was sufficient or more than sufficient

^a^Higher than high school education level percentage (%).

^b^Employed percentage (%).

### Characteristics of the Participants Recruited Online Versus Offline

Statistical analysis revealed that participants recruited through online methods tended to be younger than those recruited offline, with a mean age of 39.64 years compared with 47.4 years, respectively (see Table 3). Additionally, a higher proportion of participants recruited online had attained education beyond high school (mean 91.31%) compared with those recruited offline (mean 83.2%). However, these differences were not statistically significant (*P*=.27 for age and *P*=.24 for education level). Additionally, due to incomplete reporting of participant characteristics such as occupation, religious affiliation, marital status, gender, and household income in some studies, it was not feasible to conduct comparative analyses of these variables between participants recruited through online versus offline methods.

**Table 3:** Characteristics of the participants recruited online versus offline.

Group			Online		Offline
**Age**					
	Mean, %	39.65		47.40	
	*P* value	.27		N/A^a^	
**Education level**					
	Mean, %	91.32		83.20	
	*P* value	.24		N/A	

^a^N/A: not applicable.

## Discussion

### Principal Findings

This systematic review examined recruitment strategies and research platforms used to engage Asian Americans in online research. The findings offer valuable insights for researchers aiming to recruit Asian American participants, highlighting effective recruitment methods and digital platforms. These strategies may serve as a practical guide for enhancing participant engagement and improving representation in future studies involving this population.

It is noteworthy that most of the included studies collecting data online relied exclusively on online recruitment methods. This may be attributed to the greater accessibility and broader reach of online recruitment, which allows researchers to engage a wider and more diverse population [39]. Among the various online recruitment strategies, the use of social media groups emerged as the most commonly employed method. This may be related to recruitment efficiency. Compared with other methods, recruiting participants through social media groups can be more cost-effective [40], eliminating the need to print and distribute flyers, purchase online advertisements, or pay for survey market services. It may also be more time-efficient [41], as it avoids the need for individually contacting potential participants via email to assess their interest. In addition, social media groups are valuable resources for recruiting participants because they often have specific membership criteria. Typically, individuals must meet certain requirements to join these groups, which helps researchers efficiently identify and target their intended populations. This reduces the time and effort needed to screen participants. Furthermore, recruitment through social media groups has been reported to yield higher enrollment rates and require fewer study resources [42]. This approach may be especially beneficial for researchers with limited resources. However, it is important to acknowledge the potential for bias when recruiting solely from social media groups. As membership in these groups is typically voluntary and based on shared interests, participants may be more motivated or health conscious than others in the broader target population, potentially limiting the generalizability of the findings.

Among the 18 studies, 6 utilized offline recruitment methods. Compared with online recruitment, previous research has shown that offline methods are associated with higher retention rates for follow-up questionnaires [43,44]. Therefore, when a study requires the collection of multiple questionnaires or longitudinal data over time, offline recruitment may be a preferable option due to its potential for better participant retention. Among the offline recruitment methods, community-based recruitment was the most frequently used. This may be attributed to the convenience and accessibility offered by established community networks, which often provide a high concentration of eligible participants within a specific geographic or cultural area. Moreover, community members may exhibit a heightened sense of altruism and willingness to support researchers who share similar cultural backgrounds, thereby facilitating engagement and participation. This approach can facilitate efficient participant recruitment within a relatively short time frame [45]. Moreover, community partners often possess a deep understanding of their members’ needs and maintain long-standing, trusting relationships with the community [45]. Such partnerships can help overcome barriers to participation and foster trust in the research recruitment process [45].

In addition, the most frequently used online study platform was self-designed project websites. Compared with other online study platforms, self-designed project websites provided greater flexibility to include relevant information on study projects. They may also yield higher data quality related to users’ attention, comprehension, honesty, and reliability [46]. Furthermore, self-designed project websites provide greater control over data collection, storage, transmission, and use [47]. However, their use may also pose challenges such as data security concerns, high costs, and the need for dedicated personnel and technical consultants for support [47].

For the included studies that collected data online, the most frequently collected demographic characteristics of participants were age, education level, occupation, gender, and marital status. Results showed that participants engaged in the online studies tended to be in middle adulthood and had an education level higher than high school. This may be related to the online data collection method, which requires participants to have a certain level of education to be capable of using the internet and accessing it.

Lastly, compared with participants recruited offline, those recruited online tended to be younger and more educated [11] in the included studies. Although these differences were not statistically significant, they may still warrant special attention from researchers when considering potential sample selection bias—particularly in studies aiming to generalize findings to a broader target population.

### Limitations

This study has some limitations. First, due to unreported data in the included studies, it was not feasible to report certain participant characteristics. Additionally, when comparing the characteristics of participants recruited online versus offline, only 2 studies used offline recruitment methods exclusively. The small sample size of the included studies may underestimate heterogeneity and introduce bias into the results [48,49]. Second, we included only studies published in English and conducted exclusively among Asian Americans, which may limit the generalizability of our findings to other countries or populations. Third, although we aimed to conduct a comprehensive review and analysis of Asian American participant recruitment, important details such as participants’ ethnicity, recruitment materials, and recruitment rates were not reported in the included studies, preventing further analysis of these variables.

An important limitation of this study is the inability to disaggregate and compare recruitment practices across the diverse ethnic subgroups within the Asian American population. This gap is particularly significant given that existing literature suggests recruitment barriers and facilitators can vary markedly among subgroups such as Chinese, Filipino, Korean, Vietnamese, and South Asian Americans. Factors such as cultural norms, language barriers, immigration status, and levels of digital literacy may each contribute differently to recruitment challenges. Although the included studies often lacked this level of granularity, future research should prioritize disaggregating Asian American subgroups to better understand and address the unique recruitment needs of each group.

Lastly, online studies typically carry a potential risk of being targeted by fraudsters or bots. The included studies did not describe how they managed data verification or checked participants’ identities, which limited our ability to synthesize the strategies used for data verification in online research. Despite these limitations, this study has notable strengths, including providing a systematic narrative review of participant recruitment among Asian Americans. Further data analysis on this topic also offers valuable insights for researchers aiming to successfully conduct their studies.

### Implications for Future Research and Practice

This study has several implications that can help researchers more effectively design and conduct online studies, particularly among underrepresented or multilingual populations. First, based on previous researchers’ experiences, using mixed-method recruitment—combining both online (eg, social media, email lists) and offline methods (eg, flyers, community events)—may be an effective strategy for recruiting participants into studies. This may also help decrease sample selection bias and increase recruitment efficiency by reaching individuals who may not be active online or may have limited digital literacy [43]. Second, when selecting a study platform, the choice between a self-designed platform and a commercial platform should be guided by factors such as research budget, available technical support, data security, and cultural appropriateness. For multilingual or culturally specific populations, self-designed platforms can offer greater control over language localization, culturally sensitive design, and data ownership. Lastly, recruiting participants through online or offline methods may lead to distinct differences in participant characteristics; for example, those recruited online tend to be younger and more highly educated. This pattern may extend to other multilingual or underserved groups, and researchers should interpret their results accordingly, taking into account the demographic profile of their sample.

### Conclusions

This study systematically reviewed strategies for recruiting Asian Americans to online studies. Results showed that online recruitment was the most frequently used method across the studies. Additionally, while social media groups were the predominant strategy among online recruitment methods, community recruitment was the most frequently used approach among offline methods. The most used online study platform was self-designed project websites. Among participants recruited to online studies, those in middle adulthood with education levels higher than high school tended to be more engaged. Additionally, participants recruited online tended to be younger and more educated than those recruited offline. Although this study has some limitations, as is common in systematic reviews, it provides important information to help researchers successfully conduct their studies.

## Data Availability

All data discussed in this manuscript are available in public databases.

## Appendix

Multimedia Appendix 1 PRISMA (Preferred Reporting Items for Systematic Reviews and Meta-Analyses) 2020 checklist.

Multimedia Appendix 2 Full search strategy.

Multimedia Appendix 3 Evidence appraisal form (etiology cross section).

Multimedia Appendix 4 Checklist for Quasi-Experimental Studies.

Multimedia Appendix 5 CASP (Critical Appraisal Skills Programme) Randomized Controlled Trial checklist.

Multimedia Appendix 6 CASP (Critical Appraisal Skills Programme) Qualitative Studies checklist.

## Abbreviations

CASP: Critical Appraisal Skills Programme
GRADE: Grading of Recommendations Assessment, Development and Evaluation
HIPAA: Health Insurance Portability and Accountability Act
ICAA: Internet Communities for Asian Americans
ICEM: Internet Communities for Ethnic Minorities
JBI: Joanna Briggs Institute
MeSH: Medical Subject Headings
MTurk: Mechanical Turk
PRISMA: Preferred Reporting Items for Systematic Reviews and Meta-Analyses
REDCap: Research Electronic Data Capture

## References

[1] Asian American Health. Office of Minority Health.

[2] Budiman A, Ruiz N (2021). Key facts about Asian Americans, a diverse and growing population. Pew Research Center.

[3] (2024). Asian population by state 2024. Data Pandas.

[4] Passel J, Cohn D (2008). U.S. population projections: 2005-2050. Pew Research Center.

[5] Ðoàn Lan N, Takata Y, Sakuma KK, Irvin VL (2019). Trends in clinical research including Asian American, Native Hawaiian, and Pacific Islander participants funded by the US National Institutes of Health, 1992 to 2018. JAMA Netw Open.

[6] Obra JK, Lin B, Đoàn Lan N, Palaniappan L, Srinivasan M (2021). Achieving equity in Asian American healthcare: critical issues and solutions. J Asian Health.

[7] Liu Y, Elliott A, Strelnick H, Aguilar-Gaxiola S, Cottler LB (2019). Asian Americans are less willing than other racial groups to participate in health research. J Clin Transl Sci.

[8] Chee W, Ryu S, Quan J, Kim D, Im E (2025). Why is it difficult to recruit/retain Asian American family caregivers in a virtual intervention?. West J Nurs Res.

[9] George S, Duran N, Norris K (2014). A systematic review of barriers and facilitators to minority research participation among African Americans, Latinos, Asian Americans, and Pacific Islanders. Am J Public Health.

[10] UyBico SJ, Pavel S, Gross CP (2007). Recruiting vulnerable populations into research: a systematic review of recruitment interventions. J Gen Intern Med.

[11] Park H, Shah MM (2015). Do different recruitment methods reach different Asian demographics?. Survey Practice.

[12] Wong CK, Horn-Ross PL, Gee GC, Shariff-Marco S, Quach T, Allen L, Bautista R, La Chica Patricia Quema, Tseng W, Chang P, Clarke CA, Yang J, Le GM, Canchola A, Irwin ML, Lee SS, Gomez SL (2016). Strategies for recruiting representative samples of Asian Americans for a population-based case-control study. J Epidemiol Community Health.

[13] Valdez RS, Guterbock TM, Thompson MJ, Reilly JD, Menefee HK, Bennici MS, Williams IC, Rexrode DL (2014). Beyond traditional advertisements: leveraging Facebook's social structures for research recruitment. J Med Internet Res.

[14] Im E, Kim S, Xu S, Lee C, Hamajima Y, Inohara A, Chang K, Chee E, Chee W (2020). Issues in recruiting and retaining Asian American breast cancer survivors in a technology-based intervention study. Cancer Nurs.

[15] Quan H, Wang F, Schopflocher D, Norris C, Galbraith PD, Faris P, Graham MM, Knudtson ML, Ghali WA (2006). Development and validation of a surname list to define Chinese ethnicity. Med Care.

[16] Davern M, McAlpine D, Ziegenfuss J, Beebe TJ (2007). Are surname telephone oversamples an efficient way to better understand the health and healthcare of minority group members?. Med Care.

[17] Kim J, Lauderdale D, Shin H, Lee Y (2013). Surname sampling. Field Methods.

[18] Alolod GP, Gardiner HM, Blunt R, Yucel RM, Siminoff LA (2023). Organ donation willingness among Asian Americans: results from a national study. J Racial Ethn Health Disparities.

[19] Im E, Chee W (2005). A descriptive internet survey on menopausal symptoms: five ethnic groups of Asian American university faculty and staff. J Transcult Nurs.

[20] Im E, Chee W, Seung Hee Lee (2010). Subethnic differences in the menopausal symptom experience of Asian American midlife women. J Transcult Nurs.

[21] Le TP, Bradshaw BT, Pease M, Kuo L (2022). An intersectional investigation of Asian American men's muscularity-oriented disordered eating: associations with gendered racism and masculine norms. Eat Disord.

[22] Lingala B, Li S, Wysong A, Truong AK, Kim D, Chang ALS (2014). Low rate of dermatology outpatient visits in Asian-Americans: an initial survey study for associated patient-related factors. BMC Dermatol.

[23] Pan S, Yang C, Tsai J, Dong C (2021). Experience of and worry about discrimination, social media use, and depression among Asians in the United States during the COVID-19 pandemic: cross-sectional survey study. J Med Internet Res.

[24] Rummo PE, Ali SH, Kranick J, Thorpe LE, Yi SS (2023). Online grocery shopping behaviors and attitudes among Asian Americans. J Immigr Minor Health.

[25] Yang C, Tsai J, Pan S (2020). Discrimination and well-being among Asians/Asian Americans during COVID-19: the role of social media. Cyberpsychol Behav Soc Netw.

[26] Yu N, Pan S, Yang C, Tsai J (2020). Exploring the role of media sources on COVID-19-related discrimination experiences and concerns among Asian people in the United States: cross-sectional survey study. J Med Internet Res.

[27] Wang K, Chu JN, Oh DL, Shariff-Marco S, Allen L, Kuo M, Wong C, Bui H, Chen J, Li FM, Ma C, Truong A, Gomez SL, Nguyen TT, Tsoh JY (2024). Correlates of supportive care needs among Asian Americans with colorectal, liver, or lung cancer from a web-based patient navigation portal intervention: the Patient COUNTS study. Cancer Rep (Hoboken).

[28] Chee W, Lee Y, Im E, Chee E, Tsai H, Nishigaki M, Yeo SA, Schapira MM, Mao JJ (2017). A culturally tailored internet cancer support group for Asian American breast cancer survivors: a randomized controlled pilot intervention study. J Telemed Telecare.

[29] Chee W, Kim S, Tsai H, Liu J, Im E (2020). Effect of an online physical activity promotion program and cardiovascular symptoms among Asian American women at midlife. Comput Inform Nurs.

[30] Fang L, Schinke SP, Cole KCA (2010). Preventing substance use among early Asian-American adolescent girls: initial evaluation of a web-based, mother-daughter program. J Adolesc Health.

[31] Fang L, Schinke SP (2013). Two-year outcomes of a randomized, family-based substance use prevention trial for Asian American adolescent girls. Psychol Addict Behav.

[32] Im E, Kim S, Lee C, Chee E, Mao JJ, Chee W (2019). Decreasing menopausal symptoms of Asian American breast cancer survivors through a technology-based information and coaching/support program. Menopause.

[33] Im E, Kim S, Ji X, Park S, Chee E, Chee W, Tsai H (2017). Improving menopausal symptoms through promoting physical activity: a pilot web-based intervention study among Asian Americans. Menopause.

[34] Im E, Ko Y, Hwang H, Chee W, Stuifbergen A, Lee H, Chee E (2012). Asian American midlife women's attitudes toward physical activity. J Obstet Gynecol Neonatal Nurs.

[35] Im E, Lee B, Chee W (2010). Shielded from the real world: perspectives on internet cancer support groups by Asian Americans. Cancer Nurs.

[36] Kachabian S, Seyedmajidi S, Tahani B, Naghibi Sistani MM (2024). Effectiveness of educational strategies to teach evidence-based dentistry to undergraduate dental students: a systematic review. Evid Based Dent.

[37] Al-Dirini RMA, Thewlis D, Paul G (2012). A comprehensive literature review of the pelvis and the lower extremity FE human models under quasi-static conditions. Work.

[38] Kharel M, Sakamoto JL, Carandang RR, Ulambayar S, Shibanuma A, Yarotskaya E, Basargina M, Jimba M (2022). Impact of COVID-19 pandemic lockdown on movement behaviours of children and adolescents: a systematic review. BMJ Glob Health.

[39] Iflaifel M, Hall CL, Green HR, Willis A, Rennick-Egglestone S, Juszczak E, Townsend M, Martin J, Sprange K (2023). Widening participation - recruitment methods in mental health randomised controlled trials: a qualitative study. BMC Med Res Methodol.

[40] Christensen T, Riis AH, Hatch EE, Wise LA, Nielsen MG, Rothman KJ, Sørensen Henrik Toft, Mikkelsen EM (2017). Costs and efficiency of online and offline recruitment methods: a web-based cohort study. J Med Internet Res.

[41] Brøgger-Mikkelsen Mette, Ali Z, Zibert J, Andersen A, Thomsen S (2020). Online patient recruitment in clinical trials: systematic review and meta-analysis. J Med Internet Res.

[42] Brøgger-Mikkelsen Mette, Ali Z, Zibert JR, Andersen AD, Thomsen SF (2020). Online patient recruitment in clinical trials: systematic review and meta-analysis. J Med Internet Res.

[43] Bajardi P, Paolotti D, Vespignani A, Eames K, Funk S, Edmunds WJ, Turbelin C, Debin M, Colizza V, Smallenburg R, Koppeschaar C, Franco AO, Faustino V, Carnahan A, Rehn M, Merletti F, Douwes J, Firestone R, Richiardi L (2014). Association between recruitment methods and attrition in internet-based studies. PLoS One.

[44] Lane TS, Armin J, Gordon JS (2015). Online recruitment methods for web-based and mobile health studies: a review of the literature. J Med Internet Res.

[45] Horowitz CR, Brenner BL, Lachapelle S, Amara DA, Arniella G (2009). Effective recruitment of minority populations through community-led strategies. Am J Prev Med.

[46] Peer E, Rothschild D, Gordon A, Evernden Z, Damer E (2022). Data quality of platforms and panels for online behavioral research. Behav Res Methods.

[47] Avidan A, Weissman C, Sprung CL (2005). An internet web site as a data collection platform for multicenter research. Anesth Analg.

[48] Valentine Jc, Pigott Td, Rothstein Hr (2010). How many studies do you need?. Journal of Educational and Behavioral Statistics.

[49] Kontopantelis E, Springate D, Reeves D (2013). A re-analysis of the Cochrane Library data: the dangers of unobserved heterogeneity in meta-analyses. PLoS One.

