# Supplementary material for: Recruiting Asian Americans for Online Studies: Methodological Systematic Review
Source: J Med Internet Res. 2025 Jul 3;27:e71765. doi: 10.2196/71765 (PMC12271960; doi:10.2196/71765)
Supplement: Multimedia Appendix 2 [file jmir_v27i1e71765_app2.docx]

**Recruiting Asian Americans for Online Studies: A Methodological Systematic Review**

**Full Search Strategy**

**Search Date:** February 22, 2024
**Databases Searched:** PubMed, CINAHL, Ovid
**Search Purpose:** To identify studies focused on recruitment strategies for Asian Americans in online or web-based research studies.

**1. PubMed Search Strategy**

1. **Search Type:** MeSH Term Search
2. **Search Terms Used:** ("Internet"[MeSH Terms]) AND ("Asian Americans"[MeSH Terms])
3. **Boolean Operators Used:** AND
4. **Use of Quotations:** Standard PubMed MeSH syntax was used.
5. **Filters or Limits Applied:** No limits were applied regarding publication date, language, or publication type during the search phase.
6. **Search String:** "Internet"[MeSH Terms] AND "Asian Americans"[MeSH Terms]

**2. CINAHL Search Strategy**

1. **Search Type:** Title Search
2. **Search Terms Used:** (internet OR web-based OR online) AND (Asian Americans OR Asian American)
3. **Boolean Operators Used:** OR, AND
4. **Use of Quotations:** Quotation marks were not used; searches were conducted using exact words in the title field.
5. **Filters or Limits Applied:** No date or language restrictions were applied.
6. **Search String:** TI (internet OR web-based OR online) AND TI (Asian Americans OR Asian American)

**3. Ovid (PsycINFO) Search Strategy**

1. **Search Type:** Title Search
2. **Search Terms Used:** (internet OR web-based OR online) AND (Asian Americans OR Asian American)
3. **Boolean Operators Used:** OR, AND
4. **Use of Quotations:** No quotations used; search terms entered as keywords in the title field.
5. **Filters or Limits Applied:** No filters on publication date or type.
6. **Search String:** TI (internet OR web-based OR online) AND TI (Asian Americans OR Asian American)

**4. Additional Search Method: Manual Searching---**Reference lists of included articles were reviewed to identify additional relevant studies not captured through the database searches.
