# Supplementary material for: Recruiting Asian Americans for Online Studies: Methodological Systematic Review
Source: J Med Internet Res. 2025 Jul 3;27:e71765. doi: 10.2196/71765 (PMC12271960; doi:10.2196/71765)
Supplement: Multimedia Appendix 3 [file jmir_v27i1e71765_app3.pdf]

LEGEND: Evidence Appraisal of a Single Study

Etiology, Risk Factors, Prevalence

Cross–Sectional Study

Reviewer: **Today's Date:** **Final Evidence Level:**

Project/Topic of your Clinical Question:

Article Title:

Year: **First Author:** **Journal:**

Do the study aim/purpose/objectives and inclusion/exclusion criteria assist in answering your clinical question? ☐ Yes ☐ No ☐ Unknown

- Study Aim/Purpose/Objectives:
- Inclusion Criteria:
- Exclusion Criteria:

Is a cross-sectional study congruent with the author's study aim, purpose, or objectives above? ☐ Yes ☐ No ☐ Unknown

When reading the bolded questions, consider the bulleted questions to help answer the main question.

If you are uncertain of your skills in evidence evaluation, please consult a local evidence expert for assistance:

- CCHMC Evidence Experts

Unfamiliar terms can be found in the LEGEND Glossary.

Validity *Are the results of the Cross-Sectional Study valid?*

1.	**Are the study methods clearly described and appropriate for the question?**<br>• Is the setting clearly described and appropriate?<br>• Was there a representative sample of patients at a well-defined point in the course of the condition of interest?<br>• Is the sample population clearly described and sufficient?	☐ Yes ☐ No ☐ Unknown
2.	**Were the participants similar** *(homogeneous)* **with respect to known factors of interest** *(e.g., demographic, exposure, risk, treatment, or etiology)***?**	☐ Yes ☐ No ☐ Unknown
3.	**Were objective and unbiased criteria used to measure the variable of interest?**<br>• Was the variable of interest quantifiable and precisely measurable?<br>• Were instruments used to measure the variable of interest tested to be valid and reliable?	☐ Yes ☐ No ☐ Unknown
4.	**Was there freedom from conflict of interest?**<br>• Sponsors, Funding Agency, Investigators	☐ Yes ☐ No ☐ Unknown

Comments on Study Validity:

Reliability *Are these valid study results important?*

5.	**Did the study have a sufficiently large sample size?**<br>• Was a power analysis described?<br>• Did the sample size achieve or exceed that resulting from the power analysis?<br>• Did each subgroup also have sufficient sample size *(e.g., at least 6 to 12 participants)*?	☐ Yes ☐ No ☐ Unknown
6.	**Were the statistical analysis methods appropriate?**<br>• Were the statistical analysis methods clearly described?<br>• If subgroups were evaluated, was a statistical adjustment made for the differences?	☐ Yes ☐ No ☐ Unknown

© 2006-2024 Children's Hospital Medical Center. All Rights Reserved. *Information available at* *www.cincinnatichildrens.org/evidence.*

CC BY-NC-SA This work may be licensed under Creative Commons Attribution-NonCommercial-ShareAlike 4.0 International.

7. What were the main results of the study? *(e.g., Helpful data: Page #, Table #, Figures, Graphs)*
- ***For an Etiology Study:*** How strong is the association/correlation between known factors and the variable of interest?
- ***For a Prevalence Study:*** What is the rate? *(e.g., number per population)*
- What were the measures of statistical uncertainty *(e.g., precision)*?
  (Were the results presented with Confidence Intervals or Standard Deviations?)

8. Were the results statistically significant? ☐ Yes ☐ No ☐ Unknown
Note: *This question may not be applicable in all prevalence studies.*

9. Were the results clinically significant? ☐ Yes ☐ No ☐ Unknown
- If potential confounders were identified, were they discussed in relationship to the results?

Comments on Study Reliability:

Applicability

Can I apply these valid, important study results to my population?

10. Can the results be applied to my population of interest? ☐ Yes ☐ No ☐ Unknown
- Is the setting of the study applicable to my population of interest?
- Were the participants in this study similar to my population of interest?
- Does the variable of interest apply to my population or question of interest?

11. Are my patient's and family's values and preferences satisfied by the knowledge gained from this study *(such as outcomes considered)***?** ☐ Yes ☐ No ☐ Unknown

12. Would you include this study/article in development of a care recommendation? ☐ Yes ☐ No ☐ Unknown

Comments on Study Applicability:

Additional Comments or Conclusions *("Take-Home Points")*

Quality Level / Evidence Level

- *Consider each "No" answer and the degree to which this limitation is a threat to the validity of the results, then check the appropriate box to assign the level of quality for this study/article.*
- *Consider an "Unknown" answer to one or more questions as a similar limitation to answering "No," if the information is not available in the article.*

The Evidence Level is:

	Etiology / Risk Factors	Prevalence
**Good Quality Cross-Sectional Study**	☐ 4a	☐ 3a
**Lesser Quality Cross-Sectional Study**	☐ 4b	☐ 3b

☐ **Not Valid, Reliable, or Applicable**

© 2006-2024 Children's Hospital Medical Center. All Rights Reserved. *Information available at* www.cincinnatichildrens.org/evidence.
BY-NC-SA This work may be licensed under Creative Commons Attribution-NonCommercial-ShareAlike 4.0 International.

LEGEND: Evidence Appraisal of a Single Study

Etiology, Risk Factors, Prevalence

Cross–Sectional Study

Table of Evidence Levels

DOMAIN OF CLINICAL QUESTION	TYPE OF STUDY / STUDY DESIGN														
	Systematic Review<br>Meta–Analysis	RCT +	CCT +	Cohort<br>– Prospective	Cohort<br>– Retrospective	Case – Control	Cross – Sectional	Descriptive Study<br>Epidemiology<br>Case Series	Mixed Methods Study	Decision Analysis<br>Economic Analysis<br>Computer Simulation	Guidelines	Case Reports<br>N-of-1 Study	Bench Study	Published Expert Opinion	Local Consensus<br>Published Abstracts
Etiology / Risk Factors	1a<br>1b	2a<br>2b	3a<br>3b	3a<br>3b	4a<br>4b	4a<br>4b	4a<br>4b	4a<br>4b	2/3/4<br>a/b	5a<br>5b	5a<br>5b	5a<br>5b	5a<br>5b	5a<br>5b	5
Prevalence	1a<br>1b					2a<br>2b	3a<br>3b	4a<br>4b			5a<br>5b	5a<br>5b	5a<br>5b	5a<br>5b	5

+ RCT = Randomized Controlled Trial; CCT = Controlled Clinical Trial

Development for this appraisal form is based on:

1. Guyatt, G.; Rennie, D.; Evidence-Based Medicine Working Group.; and American Medical Association.: Users' guides to the medical literature : a manual for evidence-based clinical practice. *Users' guides to the medical literature : a manual for evidence-based clinical practice:* "JAMA & archives journals." Chicago, IL, 2002
2. Melnyk, B. M. and E. Fineout-Overholt (2005). Evidence-based practice in nursing & healthcare : a guide to best practice. Philadelphia, Lippincott Williams & Wilkins.
3. Lohr, K. N. and T. S. Carey (1999). "Assessing "best evidence": issues in grading the quality of studies for systematic reviews." Joint Commission Journal on Quality Improvement 25(9): 470-9.
4. Fineout-Overholt, E. and L. Johnston (2005). "Teaching EBP: asking searchable, answerable clinical questions." Worldviews Evid Based Nurs 2(3): 157-60.
5. Jerosch-Herold, C. (2005). "An evidence-based approach to choosing outcome measures: a checklist for the critical appraisal of validity, reliability and responsiveness studies." British Journal of Occupational Therapy 68(8): 347-53.
6. Phillips, et al: Oxford Centre for Evidence-based Medicine Levels of Evidence, 2001. Last accessed Nov 14, 2007 from http://www.cebm.net/index.aspx?o=1025.
7. Fineout-Overholt and Johnston: Teaching EBP: asking searchable, answerable clinical questions. *Worldviews Evid Based Nurs,* 2(3): 157-60, 2005.
8. Clark, E., Burkett, K., & Stanko-Lopp, D. (2009, Dec). Let Evidence Guide Every New Decision (LEGEND): an evidence evaluation system for point-of-care clinicians and guideline development teams [Cincinnati Children's LEGEND development]. J Eval Clin Pract, 15(6), 1054-1060.

© 2006-2024 Children's Hospital Medical Center. All Rights Reserved. *Information available at www.cincinnatichildrens.org/evidence.*

BY-NC-SA This work may be licensed under Creative Commons Attribution-NonCommercial-ShareAlike 4.0 International.
